# Supplementary material for: Combined [18F]-FDG PET-MR imaging for monitoring small bowel crohn’s disease
Source: Eur J Nucl Med Mol Imaging. 2025 Aug 26;53(2):1234–45. doi: 10.1007/s00259-025-07524-4 (PMC12830450; doi:10.1007/s00259-025-07524-4)
Supplement: Supplementary file 1 — Supplementary Material 1 [file 259_2025_7524_MOESM1_ESM.docx]

**Combined [^18^F]-FDG PET-MR Imaging for monitoring small bowel Crohn’s disease**

**Juho Mattila PhD^1^, Johanna Kallio PhD^2^, Eliisa Löyttyniemi MSc^3^, Pirjo Nuutila Prof.^4^, Jukka Koffert PhD^1^**

^1^Department of Gastroenterology, Turku University Hospital, P.O. Box 52, 20521 Turku, Finland, +358407188271, juho.mattila@varha.fi, ^2^Department of Radiology, Turku University Hospital, Turku, Finland, ^3^Department of Biostatistics, University of Turku, Turku, Finland, ^4^Turku PET Center, University of Turku, Turku, Finland

Corresponding author: Juho Mattila

Keywords: crohn’s disease, IBD, diagnostics, PET-MRI, FDG

**Abstract**

Crohn’s disease (CD) is a chronic and relapsing inflammatory disease of the gastrointestinal tract. Diagnostics and follow-up are difficult in small bowel, that can be only partially evaluated by conventional endoscopy. Combined positron emission tomography magnetic resonance enterography (PET-MRE) has shown potential in diagnosing small bowel CD, but its role in monitoring treatment response has not been previously established. This study aimed to evaluate whether PET-MRE can be used to assess the efficacy of medical therapy. We hypothesized that standardized uptake values (SUV) in inflamed small bowel segments would decrease following initiation of standard therapy.

A total of 35 volunteer patients with clinically suspected small bowel CD were recruited. All patients underwent ileocolonoscopy and laboratory testing, followed by [¹⁸F]-FDG PET-MRE. CD diagnosis was confirmed by small bowel capsule endoscopy. Clinicians initiated treatment based on standard diagnostics, blinded to the PET results. Eighteen patients completed follow-up [¹⁸F]-FDG PET-MRE at three months. Maximum SUV (SUV_Max_) was measured in the small intestine and compared with MRE findings.

The median SUV_Max_ decreased significantly from baseline to follow-up (3.2 vs. 2.1, p=0.0025). The simplified Magnetic Resonance Index of Activity (sMARIA) was also significantly lower at follow-up (p=0.001). Representatively, median fecal calprotectin declined (451 µg/g vs 163 µg/g, p=0.004). This preliminary prospective study suggests that [^18^F]-FDG PET-MRE may be a useful tool for assessing biochemical response to treatment in newly diagnosed small bowel CD.

Trial registration number: NCT06796959 (ClinicalTrials.gov). Retrospectively registered on 21.1.2025. Enrollment of first participant on 1.8.2020.

**1 Introduction**

Crohn’s disease (CD) is a debilitating chronic inflammatory disease of the gastrointestinal tract, with its global incidence continuously rising ^1^. Both the diagnostics and follow-up of small bowel CD can be challenging. Since CD often leads to complications such as strictures, fistulae and abscesses, there is an inevitable need to establish the diagnosis rapidly. Starting effective treatment early is essential, as early diagnosis and medical intervention can significantly improve the prognosis of CD ^2^. Due to remitting and relapsing course, CD often needs lifelong treatment and surveillance. ^3^

Diagnostics and monitoring of colonic CD is generally straightforward, as the affected area can be easily assessed with conventional endoscopy. In contrast, examining the small bowel is more complex. Samuel et. al. found that more than half of patients with known CD and negative ileocolonoscopy had active inflammation in the small bowel ^4^. Cross-sectional imaging such as magnetic resonance enterography (MRE) and computed tomography (CT) lack sufficient sensitivity for detecting luminal disease ^5–7^. Small bowel capsule endoscopy (SBCE) is sensitive in detecting mucosal erosions but lacks specificity, with up to 10% of healthy individuals showing mucosal breaks in the small intestine ^8^. Additionally, SBCE carries a risk of capsule retention, with a retention rate of up to 2.1%, as reported in a systematic review by Liao et al ^9^.

CD often presents with a distinct phenotype as 20-30% of the patients have a mild clinical course while up to a third have a more severe and complicated disease course ^10^. In addition to establishing a rapid diagnosis, it is important to quantify the degree of inflammation to help clinicians initiate appropriate treatment for each patient. Assessing treatment response in small bowel CD is often challenging, as clinical symptoms do not correlate well with inflammatory activity ^11^. Due to the limited data on long-term outcomes, cross-sectional imaging has been suggested as an adjunct for assessing transmural healing rather than as a solid treatment target, according to the STRIDE II- consensus paper ^12^. Research on prognostic factors has primarily focused on genetics and biomarkers ^13^. Signs of bowel damage have been linked to poor outcomes in early CT and MRE studies in patients with CD ^14^, but no such data exists for positron emission tomography (PET) imaging.

Fusion-PET-MRE using [^18^F]-FDG (18-fluorodeoxyglucose) tracer can detect increased glucose metabolism in the small bowel caused by inflammation, infection or tumors ^15,16^. [^18^F]-FDG PET and PET-CT were found to have a pooled sensitivity of 87% in detecting active inflammatory bowel disease (IBD) according to a systematic review by Treglia et. al ^17^. MRE provides superior soft tissue contrast compared to other imaging modalities, including luminal disease ^18^. Thus, combining the detailed anatomical findings from MRE PET’s ability to quantify inflammatory activity offers a unique technique and a potential tool for clinical decision-making in CD, such as grading the inflammatory activity of a stricture. [^18^F]-FDG PET-MRE has been shown to correlate with endoscopic activity in patients suspected of IBD ^19^ and can distinguish purely fibrotic structures from mixed or inflammatory strictures ^20^. Additionally, PET-MRE has been found to be superior to PET-CT in detecting extraluminal inflammation and assessing inflammatory activity in strictures in CD patients scheduled for surgery ^21^. The European Association of Nuclear Medicine (EANM) lists the indications for [^18^F]-FDG PET imaging in IBD as follows: 1) evaluation of disease extent, 2) early assessment of therapy and 3) differential diagnostics between fibrotic and inflammatory stricture ^22^. However, limited availability, lack of standardized methods, and poorly defined diagnostic thresholds have limited the use of [^18^F]-FDG PET-MRE in both the diagnostics and follow-up of small bowel CD. Furthermore, the current European Crohn’s and Colitis Organisation (ECCO) guidelines do not place a statement for or against PET imaging ^23^.

The aim of this study was to prospectively determine whether [^18^F]-FDG PET-MRE could be used in monitoring of small bowel CD, to compare its performance with MRE alone, and to assess whether it could evaluate the response to medical therapy.

**2 Materials and methods**

Volunteer patients aged 18-70 years with suspected small bowel CD were recruited from the outpatient clinic at Turku University Hospital. These patients were referred to the gastroenterology unit due to obscure diarrhea, abdominal pain, and elevated fecal calprotectin (FC) levels (>100 µg/g), or because they had a thickened bowel wall or stricture identified on abdominal CT. Prior to recruitment, the patients underwent colonoscopy with ileal biopsies. Endoscopic findings were collected and graded according to the SES-CD-score (Simple Endoscopic Score for Crohn’s disease) ^24^. Histologic findings were collected. Inclusion criteria were: clinical suspicion of CD in terminal ileum (ileal subscore of SES-CD 2 or more), suspicion of CD in small intestine in CT or gastrointestinal symptoms and unexplainedly elevated FC. Exclusion criteria were nonsteroidal anti-inflammatory drug (NSAID) use, diabetes, previously diagnosed CD, metformin-therapy and pregnancy or any other known contraindication for MRE or SBCE. Laboratory samples including serum hemoglobin (Hb), serum albumin (Alb), C-reactive protein (CRP) and FC were collected. The Phadia^®^ EliA™ (Thermo Fischer Scientific, MA, USA) FEIA (fluorimetric enzyme-linked immunoassay) was used for FC analysis. Written informed consent was obtained.

A static PET-MRE was obtained with [^18^F]-FDG tracer and gadolinium contrast agent (Dotarem ®). CD diagnosis was confirmed with SBCE, provided no stenosis was observed in the MRE sequences. SBCE findings were graded according to the CECDAI score ^25^. Patients were referred to gastroenterologists who were blinded to the PET results. The clinicians had access to endoscopy, histology, laboratory, MRE and SBCE data, and based on this information made the diagnosis of CD according to the ECCO-ESGAR guidelines ^23^ and initiated standard treatment. After 3 months, patients diagnosed with CD underwent follow-up [^18^F]-FDG PET-MRE. Medication data were collected, along with laboratory results during the follow-up period. At the time of both PET-MRE exams, the patients filled in a validated symptom questionnaire (IBD symptom index) ^26^ which ranges from 0 to 22 points^26^. (Fig 1.)

**2.1 PET imaging**

Prior to PET imaging, patients fasted for 6 hours. Fasting glucose levels were determined from a venous blood sample. To prepare the bowel, 1200 ml of diluted 3% mannitol was ingested over 45 minutes. Patients were positioned prone to enhance small bowel visualization. 4 MBq/kg (max. 300MBq) of [^18^F]-FDG was injected into the antecubital vein. A 9 min static PET sequence was obtained at least 30 minutes after injection to allow sufficient [^18^F]-FDG accumulation in small bowel wall (median 39 min). The scan range was visually selected by the investigators from scout images covering the entire small intestine, using the gallbladder as the cranial landmark.

**2.2 MRI protocol**

A 3T-PET-MR scanner (SIGNA™ PET/MR, General Electric, Boston, MA, USA) was used for all imaging. Patients received intravenous 10 mg hyoscine butylbromide (Buscopan®, Boehringer Ingelheim International GmbH, Germany) during image aquisition to reduce motion artefact form intestinal motility. Gadoterate meglumine (Dotarem®, Guerbet, France) (0,1 mmol/kg) was used as contrast agent. MRE sequences covered the same area as PET sequences. The following MRI sequences were used: first 2D breath hold (BH) fast imaging employing steady-state acquisition (FIESTA) in coronal plane, second: 2D T2-weighed single shot fast spin echo (SSFSE) in coronal and axial plane, third: 2D diffusion weighted imaging (DWI) with fat saturation in axial plane, fourth: pre- and post-contrast BH fat-saturated 3D T1 gradient echo  in coronal plane with 40 seconds post-contrast delay, fifth: post-contrast BH fat saturated 3D T1 gradient echo in axial plane.

**2.3 Image analysis**

Endoscopists, PET-MRE readers, and clinicians were blinded to each other’s results. MRE and PET sequences were co-registered using the lower edge of liver and kidneys as a reference. SUVs were calculated in multiple regions of the small intestine with corresponding anatomical localization determined from the MRE sequences. SUV is a semiquantitative variable that describes radiopharmaceutical accumulation in PET studies, reflecting tissue glucose uptake and, consequently glucose metabolism ^27^. SUV measurements were obtained from the intestinal wall using axial and coronal MR slices to confirm anatomical location. Increased uptake was visually distinguished from background activity and compared with adjacent bowel loops. All lesions with signal intensity higher than that of the liver and the adjacent bowel loops were assessed. The “Send cursor to max value” -function in AW VolumeShare 5, 11.3 (GE Healthcare 2005-2010) software was used to identify and measure the highest SUV (SUV_Max_) in each region of interest. For statistical analysis, only the lesion with highest SUV-value was included. The simplified Magnetic Resonance Index of Activity (sMARIA) score ^28^ was used to assess the degree of inflammation in the small intestine on MRE, as evaluated by an experienced abdominal radiologist. The sMARIA score was assessed from the same segment that demonstrated the highest SUV. When comparing changes in sMARIA and SUV_Max_ between the diagnostic and follow-up imaging, the same lesion was used to allow for true paired analysis.

**2.4 Statistical methods**

Categorical variables were summarized with counts and percentages, continuous variables with median and lower (Q1) and upper quartile (Q3). The mean changes over time for continuous variables (SUV_Max_, FC, CRP, Hb, Alb) were analyzed using Linear mixed model for repeated measures. Normality assumption for studentized residuals were examined and natural logarithm transformation was used to SUV_Max_, FC and CRP. Logarithm transformation (natural logarithm) was used to SUV_Max_ and FC to fulfill the assumption of normality of studentized residuals. Model based means were then back- transformed to the original scale (using formula e^mean^) to make the clinical interpretation easier. The association between SUV_Max_ change and sMARIA change categories was analyzed with one way ANOVA. When FC at follow-up was compared with group where SUV_Max_ was decreased or increased nonparametric Wilcoxon rank sum test was used. When the association between two categorical variables were examined, Fisher’s exact test was performed. To study the association between two continuous variables (like log- transformed SUV_Max,_ Hb, CRP etc) Pearson correlation was used. When comparing changes in SUV_Max_ and FC, the patients were divided into two subgroups; SUV_Max_ decreased and no decrease in SUV_Max_, or FC between the diagnostic and follow-up imaging and were analyzed using Wilcoxon rank sum test.

With logistic regression model area under ROC-curve was estimated. In addition, Youden- index was used to find an optimal diagnostic cutoff for SUV_Max_.

P-values (two-tailed) less than 0.05 were considered statistically significant. The data analysis for this paper was generated using SAS software, Version 9.4 of the SAS System for Windows (SAS Institute Inc., Cary, NC, USA)

**3 Results**

A total of 35 patients were screened for the study and underwent initial diagnostic [^18^F]-FDG PET-MRE. Patient demographics are presented in Table 1. Of these, 26 patients (75%) were diagnosed with small bowel CD while 9 patients (25%) did not have small bowel CD. The median SUV_Max_ was significantly higher in patients diagnosed with CD (3.3 [IQR 2.8-5.1]) compared to those without CD (1.9 [IQR 1.3-2.8]) p=0.0068. Follow-up [¹⁸F]-FDG PET-MRE was performed in 18 (69%) of the patients with CD approximately 3 months after the initial scan (median 109 days, IQR [91–128]. Sample images are shown in Fig 3. The median SUV_Max_ was significantly reduced at the follow-up compared to the initial imaging (3.2 [IQR 2.5-45] vs. 2.1 [IQR 1.6-3.5]), p=0.0025. Similarly, the sMARIA score was also significantly lower at the follow-up imaging (p=0.001). Median FC also decreased (451 µg/g [IQR 227-889] at baseline vs. 163 [IQR 81-332] µg/g at follow-up; p=0.004). (Fig. 2) No significant differences were observed in CRP (p=0.90), Hb (p=0.17), or Alb (p=0.59) between the diagnostic and follow-up imaging (p=0.90). Additionally, the IBD symptom index did not show a significant change (p=0.52). Table 2.

| **Table 1. Demographics** |  |
| --- | --- |
| Age (median) [IQR] | 31 [22-42] |
| Sex (M,F) [%] | 21 [60], 14 [40] |
| SES-CD (median) [IQR] | 3 [1-8] |
| Disease location (Montreal classification) (N) [%] | L1 (15) [83], L3 (3) [17] |
| Disease behavior (Montreal classification) (N) [%] | B1 (13) [72], B2 (3) [17], B3 (2) [11] |
| CECDAI (median) [IQR] | 4 [3-8] |
| Glucocorticoids before diagnostic imaging (N) [%] | 5 [28%] |
| Days between colonoscopy and diagnostic PET-MRE (median) [IQR] | 20 [14-32] |
| Days between diagnostic PET-MRE and SBCE (median) [IQR] | 43 [31-65] |
| Days between diagnostic PET-MRE and control MRE (median) [IQR] | 109 [91-128] |
| Days between diagnostic PET-MRE and start of glucocorticoids (median) [IQR] | 8 [5-14] |
| Days between diagnostic PET-MRE and start of immunosuppressants (median) [IQR] | 50 [27-75] |
| Days between diagnostic PET-MRE and start of advanced therapies (median) [IQR] | 114 [25-206] |

Table 1. Demographics of the patients who completed the study.

Interquartile range (IQR), Simplified Endoscopic Score for Crohn’s disease (SES-CD), Capsule Endoscopy Crohn’s Disease Activity Index (CECDAI), small bowel capsule endoscopy (SBCE). The Montreal classification of CD by age, disease location and behavior in terms of penetrance: L1 disease limited to small bowel, L2 disease limited to colon, L3 ileocolonic disease. B1 stands for luminal, non-penetrating disease, B2 is stricturing disease whereas B3 stands for fistulizing disease. ^29^

| **Table 2. Results** | **First diagnostic imaging** | **Follow-up imaging** | **p** |
| --- | --- | --- | --- |
| SUV_Max_ (median) [IQR] | 3.2 [2.5-4.5] | 2.1 [1.6-3.5] | 0.0025 |
| sMARIA (median) [IQR] | 1.5 [1-3.3] | 1 [0-2] | 0.001 |
| FC (µg/g) (median) [IQR] | 451 [227-899] | 163 [81-332] | 0.004 |
| CRP (mg/l) (median) [IQR] | 1.5 [1-12.8] | 2 [1-10.8] | 0.9 |
| Hb (g/l) (median) [IQR] | 142 [130-156] | 140 [133-150] | 0.17 |
| Alb (g/l) (median) [IQR] | 38.4 [37.1-40.7] | 39.8 [36.7-42.3] | 0.59 |
| Plasma fasting glucose (mmol/l) (median) [IQR] | 5.1 [4.9-5.5] | 5.2 [5.0-5.8] | 0.21 |
| IBD symptom index (median) [IQR] | 4.5 [2.3-7.8] | 3 [2.0-5.0] | 0.52 |

Table 2. Results for the patients diagnosed with small bowel CD from the first diagnostic [^18^F]-FDG PET-MRE and follow-up imaging in approximately 3 months (median 109d, IQR 91-128d). Interquartile range (IQR), maximum standardized uptake value (SUV_Max_), simplified Magnetic Resonance Index of Activity (sMARIA), fecal calprotectin (FC), C-reactive protein (CRP), hemoglobin (Hb), serum albumin (Alb), inflammatory bowel disease (IBD).

SUV_Max_ decreased in 13 (72%) patients, while 11 (61%) patients showed a change in sMARIA score between the diagnostic and follow-up imaging. The estimated difference in SUV_Max_ between patients with a two-point decrease in sMARIA and those with no change was significant:-5.61 (95%CI -10.5, -0.75; p=0.027). At the time of follow-up imaging, 8 out of 12 patients (66.7%) had normal CRP (<10mg/l), and 11 out of 18 patients (61.1%) had FC <250 µg/g. Using FC <250 as the cutoff for biochemical remission, the area under ROC-curve (AUC) for the natural logarithm of SUV_Max_ was 0.64. The highest sensitivity and specificity were observed at an SUV_Max_ of 3.1 (60% and 86%, respectively). There was no significant difference in SUV_Max_ or sMARIA between patients with FC <250 µg/g and those with >250 µg/g at the follow-up imaging (p=0.36 and p=0.62, respectively). When directly compared for predicting FC <250 µg/g at follow-up, the AUC was 0.66 for SUV_Max_ and 0.69 for sMARIA.

Laboratory tests performed later to assess longer time remission (median 407d, [IQR 316-454]) showed normalized CRP in 11 out of 13 patients (84.6%) and FC <250 µg/g in 12 out of 13 patients (92.3%). 10 out of 12 patients (83.3%) had both normal CRP and FC <250 µg/g . At the 12-month time point, using FC <250 µg/g and normal CRP as criteria for remission, AUC for SUV_Max_ measured from the follow-up PET-MRE was 0.63. The highest sensitivity and specificity were observed at SUV_Max_ cutoff of 3.3 (58.3% and 100%, respectively). However, only one patient had both elevated FC and CRP at that time.

SUV_Max_ correlated positively with FC at both the diagnostic and the follow-up imaging time points (r=0.56, p=0.017; and r=0.46, p=0.06, respectively), (Supplementary Fig. 1). Of the 13 patients (72%) who showed a decrease in SUV_Max_, 10 (77%) also exhibited a decrease in FC. One patient had FC <20 µg/g at both diagnostic and follow-up imaging, while two patients (15%) had higher FC at follow-up despite a decreased SUV_Max_. Five patients (28%) had a higher SUV_Max_ at follow-up compared to diagnostic imaging; among them, four (80%) had a decrease in FC despite the increased SUV_Max_. A positive correlation between CRP and SUV_Max_ was observed at both diagnostic and follow-up imaging (r=0.61, p=0.008; and r=0.53, p=0.07, respectively). A negative correlation was found between SUV_Max_ and both Alb and Hb at the time of diagnostic imaging (r=-0.66, p=0.005; and -0.64, p=0.004). At the follow-up, these correlations were weaker for (Alb: r=0.22, p=0.52; Hb: r=-0.30, p=0.23).

Patients who showed a decrease in SUV_Max_ between the diagnostic and follow-up imaging also had significantly lower FC at the time of follow-up (p=0.024), whereas no significant difference in FC was observed in patients with an increased SUV_Max_ at follow-up (p=0.63). At the 12-month time point, FC levels were significantly decreased in patients with a reduction in SUV_Max_ (p=0.039); however, this difference did not reach statistical significance in those without an initial decrease in SUV_Max_ (p=0.063).

Five patients (27.8%) were on glucocorticoids before the first diagnostic imaging. There was no significant difference in SUV_Max_ between CD-patients who were on glucocorticoids prior to the diagnostic imaging and those who were not (median 3.8 [IQR 2.2-3.9] vs. 3.1 [IQR 2.5-5.2], p=0.44). All the 18 patients who had a follow-up imaging, received a glucocorticoid induction between the two PET-MREs. Six of the patients (33.3%) were started on advanced therapies (infliximab or adalimumab) during the study. Seven patients (38.9%) were treated with a glucocorticoid induction followed by an immunomodulator (mercaptopurine or methotrexate). Five patients (27.8%) received only glucocorticoids. There was no significant difference in SUVs at the time of the follow-up imaging between the patients treated with glucocorticoids only and the patients treated with immunosuppressants (median 1.8 [IQR 1.7-2.0] vs 2.2 [IQR 1.8-2.7], p=0.53), nor with glucocorticoids only and with advanced therapies (median 1.8 [IQR 1.7-2.0] vs 3.2 [IQR 1.6-4.8], p=0.54), nor with glucocorticoids only to either advanced therapies or immunosuppressants (median 1.8 [IQR 1.7-2.0] vs 2.2 [IQR 1.6-3.8], p=0.44). Patients treated with advanced therapies had a significant decrease in SUV_Max_ at the follow-up imaging (median 5.5 [IQR 4.0-8.4] vs 3.2 [IQR 1.6-4.8], p=0.027), but we found no significant change in SUV_Max_ between the two PET-MREs in patients treated with immunosuppressants (median 3.1[IQR 3.0-3.4] vs 2.2 [IQR 1.8-2.7], p=0.20) or glucocorticoids (median 2.8 [IQR 2.5-3.6] vs 1.8 [IQR 1.7-2.0], p=0.24).

**4 Discussion**

SUV_Max_ decreased significantly at follow-up imaging, suggesting a response to medical treatment in patients with a newly diagnosed small bowel CD. A decrease in SUV_Max_ at three months was modestly associated with biochemical remission, defined as a decrease of FC to <250 µg/g. In this setup, SUV_Max_ cutoff at 3.1 was associated with biochemical response to medical therapy. We also showed that SUV_Max_ was higher in patients with small bowel CD compared to those without small bowel CD. These findings further support the potential of [^18^F]-FDG PET-MRE as a tool for evaluating intestinal inflammation in CD. To our knowledge, this is the first study to demostrate that [^18^F]-FDG PET-MRE may be used in the follow-up of small bowel CD to monitor response to medical treatment. A strong association between SUV_Max_ and FC was observed, with a statistically significant correlation, highlighting the potential of SUV_Max_ to quantify the degree of intestinal inflammation. The diagnostic accuracy of fusion PET-MRE was comparable to that of MRE alone when assessed using the sMARIA score. SUV_Max_ also decreased significantly in patients whose sMARIA score decreased by ≥2 points, while the difference was less clear in those with a ≥1-point decrease. Notably, a reduction in SUV_Max_ was observed in slightly more patients than a reduction in sMARIA (72% vs. 61%), which may suggest that resolution of inflammation is detectable earlier on PET imaging due to reduced glucose metabolism.

To highlight the challenges of assessing inflammation based solely on clinical and laboratory markers, we found no significant changes or correlations with CRP, Hb, Alb or the IBD symptom index. FC was selected as the primary parameter to define remission, using a cutoff value of 250  µg/g in accordance with the STRIDE-II consensus ^12^. However, the optimal threshold for FC remains somewhat controversial, with proposed cutoffs ranging from 100–250  µg/g. This is particularly relevant in luminal small bowel CD, where FC values tend to be lower. Indeed, patients with ileal CD have significantly lower FC levels than those with colonic or ileocolonic disease ^30^. Despite ongoing debate regarding the ideal FC threshold, several studies have demonstrated a correlation between FC and the SES-CD ^31^. FC is also strongly associated with findings in SBCE, more with Lewis-score than CECDAI-score ^32^. FC has been shown to correlate with clinical indices, such as the Crohn’s disease Activity Index (CDAI)^33^, although this was not observed in our study population. We found no correlation between SUV_Max_ -values or FC and the IBD symptom index, likely due to the predominance of mild luminal disease among our study population. The IBD symptom index is based solely on patient-reported outcomes and general well-being, whereas the CDAI includes physician’s assessment and laboratory parameters. It is also well documented that up to 25% of IBD patients experience irritable bowel symptoms while in remission ^34^, which may explain the lack of significant change in the IBD symptom index observed in our study. To date, FC remains the most reliable single laboratory marker for predicting remission, relapse, and response to therapy. ^31^ The absence of significant associations between Hb, CRP, or albumin and PET findings in our study likely reflects the mild, luminal disease profile of the cohort—findings that are consistent with those reported by Ahmadi et al. using [¹⁸F]-FDG PET-CT ^35^. Regarding long-term prognostic evaluation of PET-MRE, our data are limited due to the small sample size. At the 12-month follow-up, only one patient had both elevated CRP and FC; the rest were in biochemical remission, based on FC and CRP levels.

There are a few studies investigating the use of the [^18^F]-FDG tracer in PET-CT to detect active CD inflammation; however, no diagnostic threshold values have been previously established ^36^. Previous studies have been conducted either in patients with known CD or in mixed populations that include both ulcerative colitis and CD patients. SUVs measured in this study were lower than those reported in [^18^F]-FDG PET-CT studies. For example, Ahmadi et. al. reported a mean SUV_Max_ 4.77 in inflamed areas ^35^. This discrepancy could be attributed to several factors, including inferior attenuation correction in PET-MRI scanners compared to PET-CT scanners as well as differences in the time interval between [¹⁸F]-FDG injection and imaging (90 minutes in their study vs. a median of 39 minutes in ours). In our protocol, a minimum uptake period of 30 minutes was selected for the static PET scan to reduce total scan time and to improve patient comfort. Time-activity curves of the small bowel indicate that equilibrium is typically reached after 30 minutes post-injection. While a longer uptake time could potentially result in higher SUV_Max_ values—and thereby enhance the detection of changes between baseline and follow-up imaging—further research is needed to evaluate the utility of dynamic imaging in small bowel inflammation. This difference in uptake periods before scans makes comparing our SUV_Max_ levels difficult to those of previous studies with scans starting later. Establishing universal SUV cutoff values remains controversial due to variability in imaging protocols and scanner characteristics, including image acquisition time, scanner sensitivity, attenuation correction algorithms, signal-to-noise ratios, partial volume effect reduction, and dead time correction. We also hypothesize that disease severity influences SUVs, although our sample size was insufficient to draw statistically significant conclusions on this aspect. We found a statistically higher SUV_Max_ in patients with small bowel CD compared to those not diagnosed with CD, but the non-CD patients were not truly healthy controls, as they also had clinical suspicion on small bowel CD. This is a confounding factor, as those patients may have suffered from some other transient inflammation of the small bowel. A study with asymptomatic healthy volunteers compared to CD-patients would prove more beneficial data on this.

In a study by Catalano et al., the sensitivity of [¹⁸F]-FDG PET-MRI in detecting active Crohn’s disease (CD) inflammation was higher than that of MRI alone (88% vs. 80%), and its specificity was also superior (91% vs. 83%) in patients with established CD scheduled for surgery ^37^. In contrast, our study population comprised milder cases, with only one patient undergoing surgery during the follow-up period. This difference likely accounts for the lower sensitivity observed in our results. Based on these findings, we hypothesize that transmural inflammation leads to higher glucose metabolism—and thus higher [¹⁸F]-FDG uptake—than luminal inflammation. Similarly, a study by Pellino et al. reported a mean SUV of 2.0 in actively inflamed areas and 3.8 in inflamed strictures. They proposed SUV_Max_ cutoff of 2.95 to identify active inflammation, which closely aligns with our findings. Quantitative [¹⁸F]-FDG PET-MRE was shown to correlate with the inflammatory activity of strictures in CD, supporting its potential use in clinical decision-making—particularly when determining whether to escalate medical therapy or consider surgical intervention. ^21^ The role of [¹⁸F]-FDG PET imaging in monitoring treatment response has remained uncertain, mainly due to the limited number of studies, small sample sizes, and the predominant use of PET or PET-CT rather than PET-MRI ^38,39^. To date, the study by Pellino et al. is the only one to demonstrate the clinical utility of PET-MRI, and it focused on patients with known, complicated CD ^21^.

We found no significant differences in SUV_Max_ among patients receiving different medical therapies at the time of follow-up imaging. While this may suggest that the treatments were similarly effective, the study lacked sufficient statistical power to draw definitive conclusions. Furthermore, there was considerable variability in the timing between the diagnostic imaging and initiation of therapy, particularly with advanced treatments, which may have influenced the results. It is also possible that, in some cases, inflammation resolved spontaneously—a phenomenon occasionally observed in Crohn’s disease. To more conclusively establish the prognostic value of PET-MRE, a larger-scale study with patients randomized to treatment and placebo arms would be necessary.

Glucocorticoids most likely played a major role in inducing remission and contributed to the observed decreases in both SUV_Max_ and sMARIA scores. However, they also represent a potential confounding factor affecting glucose uptake measurements, as nearly one-third of patients were already receiving glucocorticoids prior to the diagnostic PET-MRE ^40^. Glucocorticoids are known to decrease the diagnostic yield of PET-imaging ^40–42^ and are commonly used to treat CD inflammation^43^. Despite this, no significant difference in SUV_Max_ was found between CD patients who were and were not on glucocorticoids prior to the diagnostic imaging. Additionally, all patients in the study had fasting glucose levels below 6.3 mmol/L, in accordance with EANM guidelines ^44^. Conditions that elevate blood glucose such as unbalanced diabetes can lead to reduced SUVs, while metformin is known to increase glucose uptake in the small bowel, potentially elevating SUVs ^45,46^. In our cohort, glucocorticoids were initiated in highly symptomatic patients at the time of diagnostic endoscopy. Immunosuppressive therapies, which typically require longer durations to induce remission, likely had limited impact on the short-term imaging outcomes observed in this study. Conversely, advanced therapies such as anti-TNF agents often elicit a more rapid response; however, in our population, these were typically initiated after the follow-up PET-MRE, with the median initiation time exceeding the interval between diagnostic and follow-up scans. This delay is consistent with clinical practice, where advanced therapies are generally reserved for patients who fail to respond to glucocorticoids or immunosuppressants.

In a previous study, we demonstrated that CD patients treated with advanced therapies or immunomodulators exhibited higher SUV_Max_ at diagnostic imaging compared to those receiving only glucocorticoids ^47^. We hypothesize that early identification of non-responders to conventional therapy through follow-up PET-MRE could facilitate timely escalation to more advanced therapies or surgical intervention. Achieving remission more rapidly would not only improve patient quality of life by alleviating symptoms but could also reduce complications requiring hospitalization or surgery and minimize the financial burden associated with prolonged use of ineffective, costly therapies. However, the use of glucocorticoids may have impacted these results, and a larger scale study would be needed to confirm these initial findings.

A key strength of this study is its prospective design. All patients underwent standardized diagnostic procedures, and the treating clinicians were blinded to the PET results to minimize bias. Clinical management was guided by laboratory values, endoscopic and MRE findings, and patient-reported symptoms, with the goal of achieving remission. The main limitation of this study is the relatively small sample size, which limits statistical power and may account for the lack of significant differences in SUV_Max_ between patients receiving different treatments. The study population largely comprised patients with mild luminal disease, with only one patient requiring surgery (ileocecal resection) during the study period. The predominance of luminal disease likely influenced the imaging outcomes, as both PET and MRE findings were relatively subtle, leading to modest changes between the diagnostic and follow-up scans. For comparison between MRE and PET findings, the sMARIA score was selected due to its simplicity, validation, and robustness as a measure of Crohn’s disease (CD) inflammation. The study experienced a notable dropout rate: nine patients were excluded due to the absence of a CD diagnosis, six declined follow-up imaging, and two were excluded due to diagnostic uncertainty, with clinicians opting for non-medicated follow-up. The median interval between colonoscopy and the initial PET-MRE was 20 days, a period considered adequate for mucosal healing at biopsy sites, minimizing the risk of biopsy-related inflammation and hence increased SUVs.

Limitations to the use of PET-MRE include limited availability, high costs, and exposure to radiation—although the radiation dose is lower compared to PET-CT, and has been further reduced with advancements in imaging technology ^18,48^. Another concern with [^18^F]-FDG is its limited specificity, as increased glucose metabolism is observed also in infections and tumors ^16^. Glucocorticoids are commonly used especially in the induction of remission in CD, which can cause challenges in interpreting PET results. A common challenge in PET imaging is also the occurrence of incidental findings; increased glucose uptake is often observed in the small bowel of asymptomatic patients undergoing scans for non-IBD-related conditions. The present study does not address this issue. However, several new radiotracers are under research. For example, [^68^Ga]Ga-FAPI^49–51^ has shown promise in detecting fibrosis in Crohn’s disease. In addition, various tracers have been explored in preclinical models, including radiolabeled fragments from monoclonal antibodies in immuno-PET^52^, ^89^Zr-labeled infliximab^53^ or [^68^Ga]Ga-DOTA-Siglec-9 for detecting vascular adhesion protein-1 (VAP-1)^54^. These developments may offer improved specificity and expand the clinical utility of PET imaging in inflammatory bowel disease.

The accuracy of [¹⁸F]-FDG PET-MRE may be limited in cases of mild, luminal small bowel Crohn’s disease. Compared to PET-CT, PET-MRE is more prone to motion artifacts due to longer image acquisition times. To mitigate these issues, breathing-gated imaging was employed, and both butylbromide and mannitol were used to achieve adequate bowel distension. Background noise in PET sequences is another confounding factor that must be considered during image interpretation; regions of increased glucose uptake should always be carefully correlated with anatomical data from the MRE sequences and compared to glucose uptake of liver. To enhance accuracy, imaging in this study was performed in a single session combining PET and MRE, rather than separate sessions. Only the lesion with the highest SUV was included in the analyses, which limits the ability to assess disease extent. The extent of disease is clinically relevant, as it influences both patient symptoms and treatment decisions. Notably, commonly used imaging scoring systems for CD such as sMARIA and the PET-MR Index ^55^, also evaluate only a single lesion. Palatka et al.^38^ proposed a global PET score that assesses total disease burden by summing the highest SUVs in each bowel segment; however, in their methodology, the entire small bowel was treated as a single segment. In contrast, endoscopic indices like SES-CD and CECDAI do evaluate disease extent across multiple segments. While measuring mean SUVs from larger volumes of interest (VOIs) could provide more information on disease extent, this approach introduces potential errors. Large VOIs inevitably include bowel contents and background activity, which may not accurately reflect inflammation in the bowel wall itself.

PET-MRE carries no risk of capsule retention as seen with in SBCE, nor the risk of perforation associated with colonoscopy, while still allowing full visualization of the small intestine. Importantly, PET-MRE can be safely used even in severe acute setting of CD ^17,21,56^. One clear advantage of PET-MRE is its ability to evaluate the entire small intestine in a single comprehensive study. By combining quantitative information on glucose metabolism from PET sequences with detailed anatomical imaging from MRE sequences, clinicians can precisely localize and assess the extent of inflammation. This facilitates targeted biopsies and supports both medical and surgical decision-making—even for small or localized lesions. In contrast, FC, although widely used for monitoring treatment response, only reflects neutrophil migration into the gastrointestinal tract as a general marker of inflammation. It does not provide any information about the anatomical location or the extent of disease. Since [¹⁸F]-FDG uptake has been shown to correlate with inflammatory activity in CD, PET-MRE can also help evaluate inflammation within fibrotic lesions ^21,37^; This distinction is critical in clinical practice: non-inflammatory fibrotic lesions are often managed surgically, whereas evidence of inflammatory activity may support escalation of medical therapy.

To our knowledge, this is the first prospective study to evaluate small bowel glucose uptake at the time of small bowel CD diagnosis and to compare it early the initiation of medical therapy in human patients. Our findings demonstrate that SUV_Max_ decreases after initiation of standard therapy for small bowel CD. We also found an association with SUV_Max_ decrease and biochemical remission suggesting an early therapeutic response. Based on these results, [¹⁸F]-FDG PET-MRE appears to be a valuable tool not only for the diagnosis of small bowel CD but also for monitoring treatment response.

**Author Contributions:**

Conceptualization, Jukka Koffert; methodology, Jukka Koffert; formal analysis, Juho Mattila, Eliisa Löyttyniemi; investigation, Juho Mattila; resources, Juho Mattila; data curation, Juho Mattila, Johanna Kallio; writing—original draft preparation, Juho Mattila; writing—review and editing, Jukka Koffert, Johanna Kallio, Eliisa Löyttyniemi, Pirjo Nuutila; visualization, Juho Mattila; supervision, Jukka Koffert, Pirjo Nuutila; project administration, Jukka Koffert; funding acquisition, Juho Mattila, Jukka Koffert, Pirjo Nuutila. All authors have read and agreed to the published version of the manuscript.

**Funding:**

This study was supported by InFlames Finnish Flagship Programme Funding, Turku University Hospital, University of Turku, the Finnish Government research funding, a grant form MARY OCH GEORG C. EHRNROOTHS STIFTELSE (grant number: 202300058), a grant from Finnish Medical Foundation (grant number: 7849) and a grant from Research Council of Finland (grant number: 347251)

**Institutional Review Board Statement**:

The study plan was approved by the committee of ethics of Hospital District of Southwest Finland, approval number ETMK 49/2019.

**Clinicaltrials:**

NCT06796959

**Informed Consent Statement:**

Written informed consent was obtained from patients participating in this study.

**Data Availability Statement:**

Study data were collected from Turku University Hospital electronic patient database. Anonymized research data sets will be preserved and made accessible through the Finnish Social Science Data Archives once the whole project is finalized. Further enquiries can be directed to the corresponding author.

**Conflicts of Interest:**

The authors declare no conflict of interest. The funders had no role in the design of the study; in the collection, analyses, or interpretation of data; in the writing of the manuscript, or in the decision to publish the results.

**Legends**


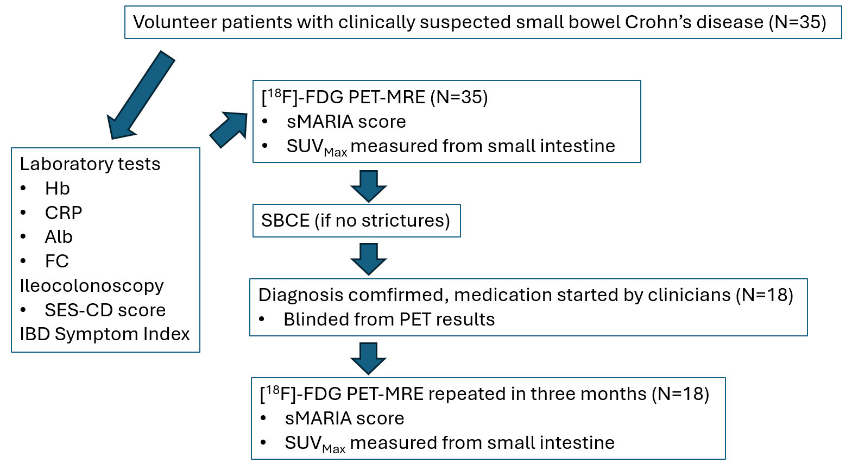


Fig 1. Study design outline. Hemoglobin (Hb), C-reactive protein (CRP), serum albumin (Alb), fecal calprotectin (FC), Simple Endoscopic Score for Crohn’s Disease (SES-CD), inflammatory bowel disease (IBD), simplified Magnetic Resonance Index of Activity (sMARIA), maximum standardized uptake value (SUV_Max_), small bowel capsule endoscopy (SBCE)


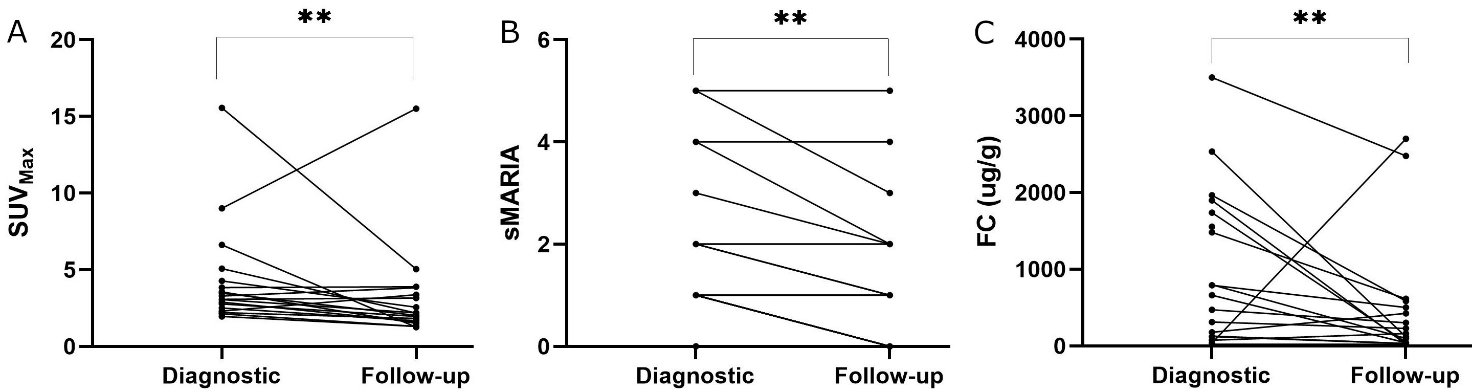


Fig 2. (A) Maximum standardized uptake value (SUV_Max_) measured in the initial diagnostic [18F]-FDG PET-MRE of the small intestine in patients diagnosed with small bowel Crohn’s disease (CD), compared to the 3-month follow-up imaging. A statistically significant decrease (p=0.0025, Linear mixed model) was observed following the initiation of medical therapy. (B) The simplified Magnetic Resonance Index of Activity (sMARIA) score, indicating CD activity in MRE, is shown for patients at diagnosis and at the 3-month follow-up. The median sMARIA score was significantly lower in the follow-up imaging (p=0.001, Wilcoxon matched-pairs signed-rank test). (C) Fecal calprotectin (FC) levels at the time of the initial diagnostic [18F]-FDG PET-MRE and at the 3-month follow-up are shown. Median FC levels were significantly reduced at follow-up (p=0.004, Linear mixed model), reflecting a biochemical response and decreased inflammation.
** p≤0.005.


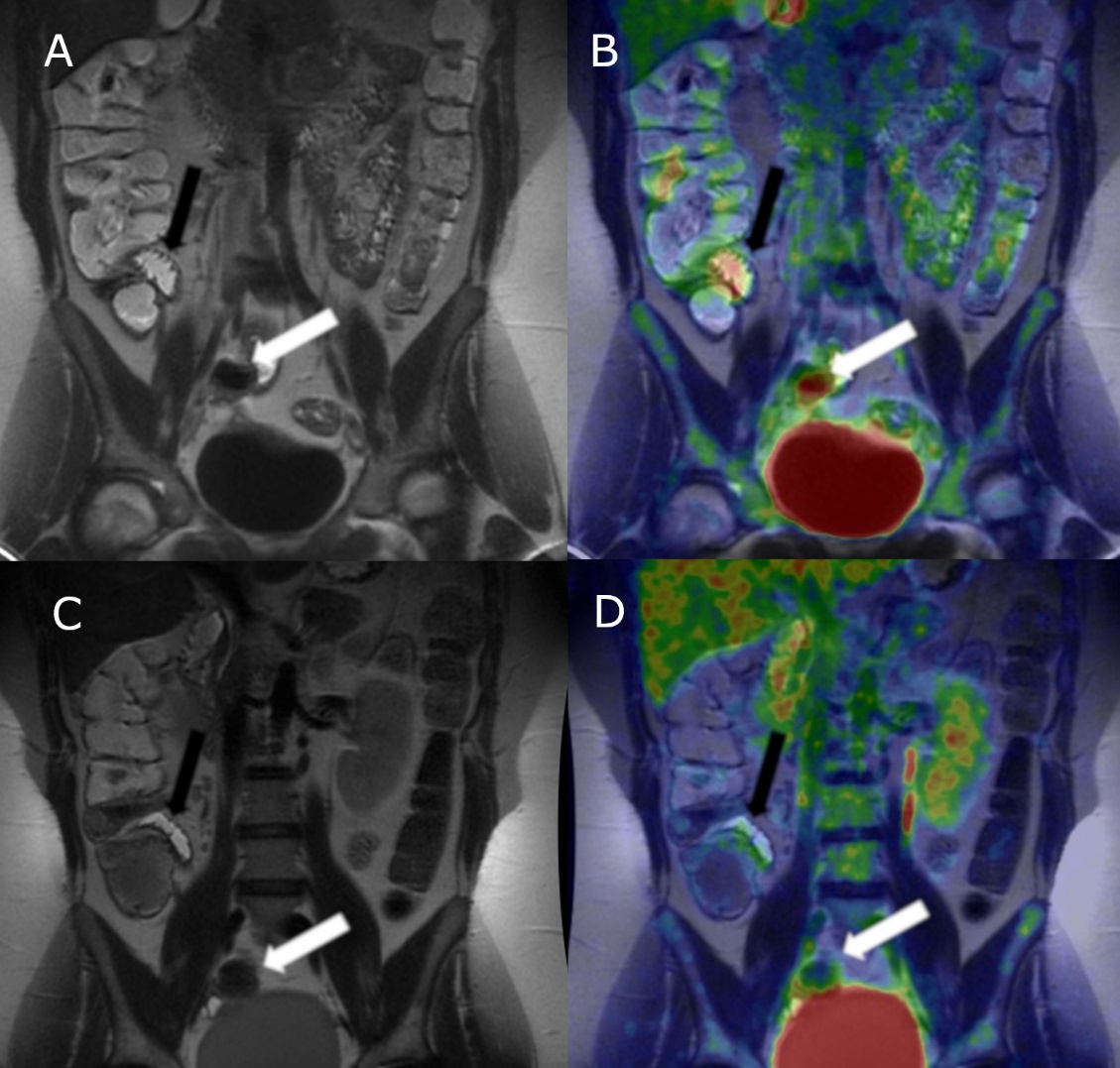


Fig 3. A 27-year-old female with small bowel CD related inflammation in terminal ileum (black arrows pointing at a distal skip lesion and white arrows pointing at a proximal skip lesion). In first diagnostic T2-weighted MRE inflammation is barely seen, sMARIA score (simplified Magnetic Resonance Index of Activity) 1 (A). In fused [^18^F]-FDG PET-MRE (B) inflammation is clearly seen in both segments, SUV_Max_ 6.6. Follow-up [^18^F]-FDG-PET-MRE was done 98 days after the diagnostic. No visible inflammation in T2-weighted MRE, sMARIA score 0 (C) and a clear decrease in inflammation is seen as [^18^F]-FDG-activity has subsided in fused [^18^F]-FDG PET-MRE, SUV_Max_ 1.3 (D).

1. Ng, S. C. *et al.* Worldwide incidence and prevalence of inflammatory bowel disease in the 21st century: a systematic review of population-based studies. *The Lancet* **390**, 2769–2778 (2017).

2. Danese, S., Fiorino, G. & Peyrin-Biroulet, L. Early intervention in Crohn’s disease: towards disease modification trials. *Gut* **66**, 2179–2187 (2017).

3. Dolinger, M., Torres, J. & Vermeire, S. Crohn’s disease. *The Lancet* **403**, 1177–1191 (2024).

4. Samuel, S. *et al.* Endoscopic Skipping of the Distal Terminal Ileum in Crohn’s Disease Can Lead to Negative Results From Ileocolonoscopy. *Clinical Gastroenterology and Hepatology* **10**, 1253–1259 (2012).

5. Costamagna, G. *et al.* A prospective trial comparing small bowel radiographs and video capsule endoscopy for suspected small bowel disease. *Gastroenterology* **123**, 999–1005 (2002).

6. Kovanlikaya, A. *et al.* PMC3645856; Magnetic resonance enterography and wireless capsule endoscopy in the evaluation of patients with inflammatory bowel disease. *Clin Imaging* **37**, 77–82 (2013).

7. Papanikolaou, N. *et al.* Contrast-enhanced magnetic resonance cholangiography versus heavily T2-weighted magnetic resonance cholangiography. *Invest Radiol* **36**, 682–686 (2001).

8. Gomollón, F. *et al.* 3rd European Evidence-based Consensus on the Diagnosis and Management of Crohn’s Disease 2016: Part 1: Diagnosis and Medical Management. *J Crohns Colitis* **11**, 3–25 (2017).

9. Liao, Z., Gao, R., Xu, C. & Li, Z. S. Indications and detection, completion, and retention rates of small-bowel capsule endoscopy: a systematic review. *Gastrointest Endosc* **71**, 280–286 (2010).

10. Lichtenstein, G. R. *et al.* ACG Clinical Guideline: Management of Crohn’s Disease in Adults. *American Journal of Gastroenterology* **113**, 481–517 (2018).

11. Cellier, C. *et al.* Correlations between clinical activity, endoscopic severity, and biological parameters in colonic or ileocolonic Crohn’s disease. A prospective multicentre study of 121 cases. The Groupe d’Etudes Therapeutiques des Affections Inflammatoires Digestives. *Gut* **35**, 231–235 (1994).

12. Turner, D. *et al.* STRIDE-II: An Update on the Selecting Therapeutic Targets in Inflammatory Bowel Disease (STRIDE) Initiative of the International Organization for the Study of IBD (IOIBD): Determining Therapeutic Goals for Treat-to-Target strategies in IBD. *Gastroenterology* **160**, 1570–1583 (2021).

13. Verstockt, B., Parkes, M. & Lee, J. C. How Do We Predict a Patient’s Disease Course and Whether They Will Respond to Specific Treatments? *Gastroenterology* **162**, 1383–1395 (2022).

14. Fiorino, G. *et al.* Prevalence of Bowel Damage Assessed by Cross-Sectional Imaging in Early Crohn’s Disease and its Impact on Disease Outcome. *J Crohns Colitis* jjw185 (2016) doi:10.1093/ecco-jcc/jjw185.

15. Yamada, S., Kubota, K., Kubota, R., Ido, T. & Tamahashi, N. High accumulation of fluorine-18-fluorodeoxyglucose in turpentine-induced inflammatory tissue. *J Nucl Med* **36**, 1301–6 (1995).

16. Boss, A. *et al.* Hybrid PET/MRI of Intracranial Masses: Initial Experiences and Comparison to PET/CT. *Journal of Nuclear Medicine* **51**, 1198–1205 (2010).

17. Treglia, G. *et al.* Diagnostic performance of Fluorine-18-Fluorodeoxyglucose positron emission tomography in patients with chronic inflammatory bowel disease: A systematic review and a meta-analysis. *J Crohns Colitis* **7**, 345–354 (2013).

18. Biondi, M. *et al.* The Role of Magnetic Resonance Enterography in Crohn’s Disease: A Review of Recent Literature. *Diagnostics* **12**, 1236 (2022).

19. Tenhami, M. *et al.* The value of combined positron emission tomography/magnetic resonance imaging to diagnose inflammatory bowel disease: a prospective study. *Acta radiol* **62**, 851–857 (2021).

20. Catalano, O. A. *et al.* Evaluation of Quantitative PET/MR Enterography Biomarkers for Discrimination of Inflammatory Strictures from Fibrotic Strictures in Crohn Disease. *Radiology* **278**, 792–800 (2016).

21. Pellino, G. *et al.* PET/MR Versus PET/CT Imaging: Impact on the Clinical Management of Small-Bowel Crohn’s Disease. *J Crohns Colitis* **10**, 277–285 (2016).

22. Abikhzer, G. *et al.* EANM/SNMMI guideline/procedure standard for [18F]FDG hybrid PET use in infection and inflammation in adults v2.0. *Eur J Nucl Med Mol Imaging* **52**, 510–538 (2025).

23. Maaser, C. *et al.* ECCO-ESGAR Guideline for Diagnostic Assessment in IBD Part 1: Initial diagnosis, monitoring of known IBD, detection of complications. *J Crohns Colitis* **13**, 144-164K (2019).

24. Daperno, M. *et al.* Development and validation of a new, simplified endoscopic activity score for Crohn’s disease: the SES-CD. *Gastrointest Endosc* **60**, 505–512 (2004).

25. Gal, E., Geller, A., Fraser, G., Levi, Z. & Niv, Y. Assessment and validation of the new capsule endoscopy Crohn’s disease activity index (CECDAI). *Dig Dis Sci* **53**, 1933–1937 (2008).

26. Puolanne, A.-M. *et al.* Rapid Fecal Calprotectin Test and Symptom Index in Monitoring the Disease Activity in Colonic Inflammatory Bowel Disease. *Dig Dis Sci* **62**, 3123–3130 (2017).

27. Kinahan, P. E. & Fletcher, J. W. Positron Emission Tomography-Computed Tomography Standardized Uptake Values in Clinical Practice and Assessing Response to Therapy. *Seminars in Ultrasound, CT and MRI* **31**, 496–505 (2010).

28. Ordás, I. *et al.* Development and Validation of a Simplified Magnetic Resonance Index of Activity for Crohn’s Disease. *Gastroenterology* **157**, 432-439.e1 (2019).

29. Satsangi, J. The Montreal classification of inflammatory bowel disease: controversies, consensus, and implications. *Gut* **55**, 749–753 (2006).

30. Gecse, K. B. *et al.* Impact of disease location on fecal calprotectin levels in Crohn’s disease. *Scand J Gastroenterol* **50**, 841–847 (2015).

31. Vernia, F. *et al.* Is fecal calprotectin an accurate marker in the management of Crohn’s disease? *J Gastroenterol Hepatol* **35**, 390–400 (2020).

32. Koulaouzidis, A., Douglas, S. & Plevris, J. N. Lewis score correlates more closely with fecal calprotectin than Capsule Endoscopy Crohn’s Disease Activity Index. *Dig Dis Sci* **57**, 987–993 (2012).

33. Sipponen, T. *et al.* Crohnʼs disease activity assessed by fecal calprotectin and lactoferrin: Correlation with Crohnʼs disease activity index and endoscopic findings. *Inflamm Bowel Dis* **14**, 40–46 (2008).

34. Fairbrass, K. M., Costantino, S. J., Gracie, D. J. & Ford, A. C. Prevalence of irritable bowel syndrome-type symptoms in patients with inflammatory bowel disease in remission: a systematic review and meta-analysis. *Lancet Gastroenterol Hepatol* **5**, 1053–1062 (2020).

35. Ahmadi, A. *et al.* Diagnostic value of noninvasive combined fluorine-18 labeled fluoro-2-deoxy-D-glucose positron emission tomography and computed tomography enterography in active Crohnʼs disease. *Inflamm Bowel Dis* **16**, 974–981 (2010).

36. Noriega-Álvarez, E. & Martín-Comín, J. Molecular Imaging in Inflammatory Bowel Disease. *Semin Nucl Med* **53**, 273–286 (2023).

37. Catalano, O. A. *et al.* Diagnostic performance of PET/MR in the evaluation of active inflammation in Crohn disease. *Am J Nucl Med Mol Imaging* **8**, 62–69 (2018).

38. Palatka, K. *et al.* The potential role of FDG PET-CT in the characterization of the activity of Crohn’s disease, staging follow-up and prognosis estimation: a pilot study. *Scand J Gastroenterol* **53**, 24–30 (2018).

39. Spier, B. J., Perlman, S. B., Jaskowiak, C. J. & Reichelderfer, M. PET/CT in the Evaluation of Inflammatory Bowel Disease: Studies in Patients Before and After Treatment. *Mol Imaging Biol* **12**, 85–88 (2010).

40. Taimen, K. *et al.* The Clinical Impact of Using ^18^ F-FDG-PET/CT in the Diagnosis of Suspected Vasculitis: The Effect of Dose and Timing of Glucocorticoid Treatment. *Contrast Media Mol Imaging* **2019**, 1–8 (2019).

41. Imfeld, S. *et al.* [18F]FDG positron emission tomography in patients presenting with suspicion of giant cell arteritis—lessons from a vasculitis clinic. *Eur Heart J Cardiovasc Imaging* **19**, 933–940 (2018).

42. Pijl, J. P., Glaudemans, A. W. J. M., Gheysens, O., Slart, R. H. J. A. & Kwee, T. C. Importance of Blood Glucose Management Before ^18^ F-FDG PET/CT in 322 Patients with Bacteremia of Unknown Origin. *Journal of Nuclear Medicine* **64**, 1287–1294 (2023).

43. Gordon, H. *et al.* ECCO Guidelines on Therapeutics in Crohn’s Disease: Medical Treatment. *J Crohns Colitis* **18**, 1531–1555 (2024).

44. Boellaard, R. *et al.* FDG PET/CT: EANM procedure guidelines for tumour imaging: version 2.0. *Eur J Nucl Med Mol Imaging* **42**, 328–354 (2015).

45. Koffert, J. *et al.* PMC5969261; Morbid obesity and type 2 diabetes alter intestinal fatty acid uptake and blood flow. *Diabetes Obes Metab* **20**, 1384–1390 (2018).

46. Di Dalmazi, G., Pagotto, U., Pasquali, R. & Vicennati, V. Glucocorticoids and Type 2 Diabetes: From Physiology to Pathology. *J Nutr Metab* **2012**, 1–9 (2012).

47. Mattila, J., Kallio, J., Löyttyniemi, E., Nuutila, P. & Koffert, J. Combined [18F]-FDG PET-MR Imaging: A promising tool for diagnostics of small bowel Crohn’s disease. *Digestive Diseases* 1–23 (2024) doi:10.1159/000542379.

48. Schmall, J. P. *et al.* Investigating Low-Dose Image Quality in Whole-Body Pediatric ^18^ F-FDG Scans Using Time-of-Flight PET/MRI. *Journal of Nuclear Medicine* **62**, 123–130 (2021).

49. Chen, L. *et al.* [68Ga]Ga-FAPI-04 PET/CT on assessing Crohn’s disease intestinal lesions. *Eur J Nucl Med Mol Imaging* **50**, 1360–1370 (2023).

50. Luo, Y. *et al.* Active uptake of 68Ga-FAPI in Crohn’s disease but not in ulcerative colitis. *Eur J Nucl Med Mol Imaging* **48**, 1682–1683 (2021).

51. Scharitzer, M. *et al.* Evaluation of Intestinal Fibrosis with ^68^ Ga-FAPI PET/MR Enterography in Crohn Disease. *Radiology* **307**, (2023).

52. Dmochowska, N. *et al.* Immuno-PET of Innate Immune Markers CD11b and IL-1β Detects Inflammation in Murine Colitis. *Journal of Nuclear Medicine* **60**, 858–863 (2019).

53. Yan, G. *et al.* Immuno-PET Imaging of TNF-α in Colitis Using ^89^ Zr-DFO-infliximab. *Mol Pharm* **19**, 3632–3639 (2022).

54. Bhowmik, A. A. *et al.* Detection of Intestinal Inflammation by Vascular Adhesion Protein-1-Targeted [68Ga]Ga-DOTA-Siglec-9 Positron Emission Tomography in Murine Models of Inflammatory Bowel Disease. *Mol Imaging Biol* **26**, 322–333 (2024).

55. Li, Y. *et al.* Assessment of Ileocolonic Inflammation in Crohn’s Disease: Which Surrogate Marker Is Better—MaRIA, Clermont, or PET/MR Index? Initial Results of a Feasibility Trial. *Journal of Nuclear Medicine* **60**, 851–857 (2019).

56. Casali, M. *et al.* State of the art of 18F-FDG PET/CT application in inflammation and infection: a guide for image acquisition and interpretation. *Clin Transl Imaging* **9**, 299–339 (2021).

**Appendix**

**MRE imaging protocol**

The following MRI sequences were used in the PET-MREs: First: 2D breath hold (BH) fast imaging employing steady-state acquisition (FIESTA) in coronal plane (4mm slice thickness; TE/TR: 1,4ms/3,7ms), second: 2D T2-weighed single shot fast spin echo (SSFSE) in coronal (4mm slice thickness, TE/TR: 90ms/3750-4615ms), and in axial plane (6mm slice thickness; TE/TR: 90ms/2100-2333ms), third: 2D diffusion weighted imaging (DWI) with fat saturation in axial plane (b-values of b50, b500 and b1000; 6mm slice thickness; minimum TE), fourth: pre- and post-contrast BH fat-saturated 3D T1 gradient echo  in coronal plane with 40 seconds post-contrast delay (2/4 mm slice thickness; minimum TE), fifth: post-contrast BH fat saturated 3D T1 gradient echo in axial plane (2/4 slice thickness; minimum TE).


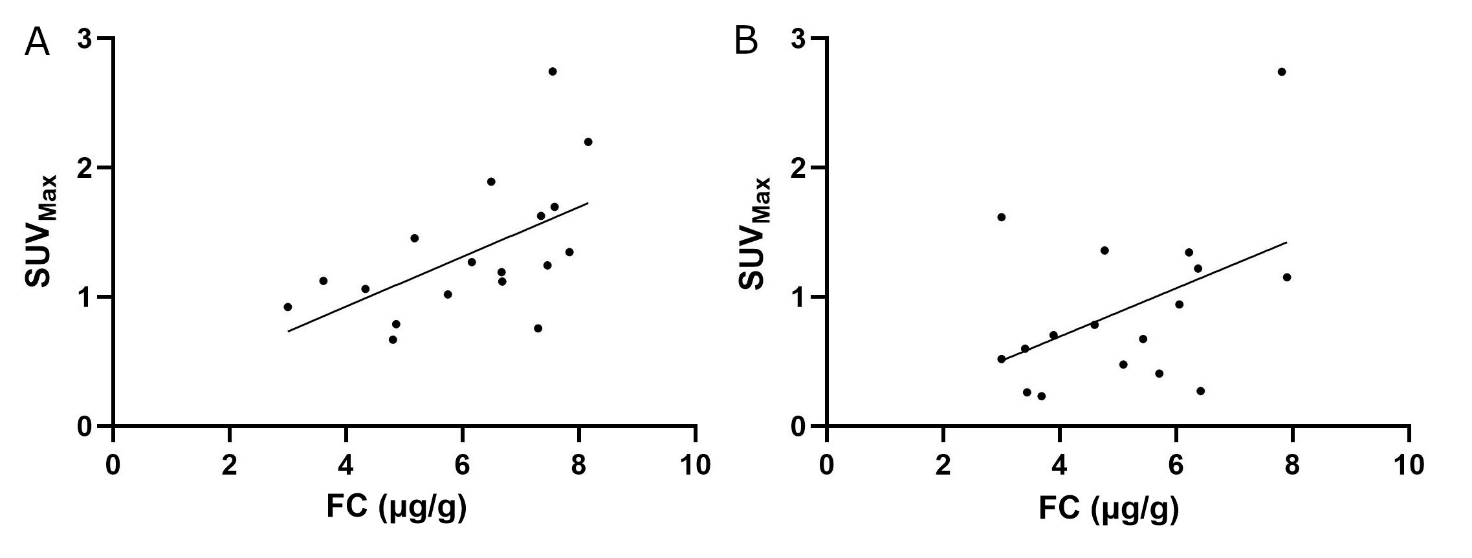


Supplementary Fig. 1

The Pearson correlations for maximum standardized uptake value (SUV_Max_) and fecal calprotectin (FC) at the diagnostic (A) (r=0.56, p=0.017) and follow-up imaging (B) (r=0.46, p=0.06) presented transformed to their natural logarithm.
